# Neural Protection Through Health Education: Early Childhood Interventions to Prevent Neurological Conditions Requiring Surgical Care

**DOI:** 10.3390/children12111529

**Published:** 2025-11-12

**Authors:** Barnabas Obeng-Gyasi, Tyler M. Nolting, Emmanuel Obeng-Gyasi, Cecilia S. Obeng

**Affiliations:** 1 Department of Neurosurgery, Allegheny Health Network, Pittsburgh, PA 15212, USA; barnabas@ahn.org; 2 Department of Health and Human Performance, Austin Peay State University, Clarksville, TN 37044, USA; noltingt@apsu.edu; 3 Department of Built Environment, North Carolina A&T State University, Greensboro, NC 27411, USA; eobenggyasi@ncat.edu; 4 Environmental Health and Disease Laboratory, North Carolina A&T State University, Greensboro, NC 27411, USA; 5 Department of Applied Health Science, Indiana University School of Public Health–Bloomington, Bloomington, IN 47405, USA

**Keywords:** early childhood education, neurosurgical prevention, traumatic brain injury, post-infectious hydrocephalus, neural tube defects, implementation science, cultural responsiveness, multi-sectoral partnerships

## Abstract

**Highlights:**

**What are the main findings?**

• Play-based and culturally responsive safety education in early childhood settings reduces traumatic brain injuries and post-infectious hydrocephalus.
• Programs involving both educators and families are more effective in building lasting protective behaviors than classroom-only approaches.

**What is the implication of the main finding?**

• Integrating neurosurgical prevention strategies into early childhood curricula can reduce preventable neurological conditions.
• Strong interdisciplinary collaboration between educators and medical specialists enhances early recognition of neurological warning signs and supports long-term child health.

**Abstract:**

Background/Objectives: This narrative review examines how developmentally appropriate safety and health education interventions in early childhood settings impact the incidence and severity of pediatric conditions requiring neurosurgical intervention, and which educational approaches most effectively promote neurological health and injury prevention among preschool-aged children. Methods: This narrative review employed a systematic literature search across medical and educational databases (ERIC, PsycINFO, MEDLINE, CINAHL, Education Source, and specialized neurosurgical sources) to identify relevant studies from 2000 to 2025. Results: Structured, play-based safety education in early childhood settings significantly reduces traumatic brain injury incidence. Programs integrating parent–educator partnerships have shown greater effectiveness in establishing protective behaviors than classroom-only approaches. Culturally responsive interventions have demonstrated specific success in high-risk communities, reducing complications from untreated hydrocephalus resulting from infections. Early childhood education can significantly impact recognition of neurological warning signs. Conclusions: Early, developmentally appropriate health education establishes protective behaviors that reduce pediatric neurosurgical cases. Implementation should prioritize experiential safety learning, recognition of neurological warning signs, and strong family–educator partnerships. Findings support integrating neurosurgical prevention strategies within early childhood curricula and developing interdisciplinary approaches connecting medical specialists with early childhood educators to reduce traumatic brain injuries, acquired hydrocephalus, and neural tube defects.

## 1. Introduction

Pediatric neurosurgical conditions pose a considerable global health burden, with epidemiological data revealing significant preventability factors across three major categories. Traumatic brain injuries (TBIs) affect 2.2% of children in the United States annually, with more than 69,000 TBI-related deaths nationwide in 2021 [1,2,3]. Post-infectious hydrocephalus is the leading cause of pediatric hydrocephalus globally, with roughly 180,000 new cases occurring annually, predominantly affecting children in low- and middle-income countries (LMICs) where neurosurgical capacity remains severely limited [4,5,6]. Neural tube defects (NTDs) occur in approximately 1–2 per 1000 live births worldwide, with rates reaching 27.4 per 10,000 births in regions lacking mandatory food fortification programs [7,8,9].

The intersection of TBI with early developmental processes has significant consequences for educational equity and long-term outcomes. Analyses of nationally representative datasets indicate that children with a history of TBI experience distinctly elevated risks of school readiness deficits, with increases ranging from 129% to 322% [3]. These disparities translate into a reduced probability of achieving kindergarten readiness from 0.424 to 0.224 [3]. Pediatric neurosurgical conditions extend beyond clinical management and directly affect educational opportunity. Injuries sustained in early childhood often lead to persistent difficulties that shape cognitive growth, school performance, and later developmental outcomes. The long-term impact is not limited to health but also influences the capacity of children to meet age-appropriate educational milestones. At the same time, emerging research on the etiology of post-infectious hydrocephalus has begun to identify specific microbial contributors, including *Paenibacillus* species, in regions with high disease burden. Such advances provide a foundation for preventive strategies that extend beyond clinical treatment, highlighting the potential of community-based educational initiatives to reduce incidence through improved awareness, hygiene practices, and early intervention [5]. The integration of implementation science and ecological systems theory provides the conceptual foundation for this review. Implementation science emphasizes how evidence-based practices can be adapted and sustained within real-world educational and healthcare systems, while ecological systems theory situates child health within the interconnected influences of family, school, community, and policy. Introducing these frameworks early helps guide the analysis of prevention strategies across multiple levels of influence. The global discussion on preventing NTDs has shifted from reliance on individual supplementation to recognizing that mandatory food fortification offers the most consistent and equitable means of reducing risk [8,9,10].

Despite growing research in each field, there remains a lack of integrative work connecting neurosurgical prevention with early educational and public health frameworks. Existing studies often address clinical management, epidemiology, or child development in isolation, leaving limited understanding of how early learning environments and community-based education can actively reduce neurosurgical risk. Few reviews have synthesized these perspectives to create a unified model of prevention spanning the individual, organizational, and policy levels. This gap underscores the need for a cross-disciplinary synthesis linking neurological health promotion with early childhood education practice.

This review examines how evidence from neurosurgery, public health, and early childhood education can be integrated to inform prevention strategies. Neurosurgical studies have focused primarily on clinical outcomes, public health research has addressed risk reduction at the population level, and educational studies have examined developmental readiness. Using an implementation science framework allows these perspectives to be considered together and underscores the need for interventions that act across multiple levels. Ecological systems theory and established public health prevention models provide the structure for this synthesis, illustrating how policies, community programs, and clinical practices can reinforce one another. This way, the review moves beyond condition-specific or discipline-specific approaches and considers how coordinated action may achieve broader and more durable preventive impact. We examine what interventions are effective and how they can be implemented with reliability and sustainability in real-world early childhood education settings. This approach is based on the understanding that population-level neuroprotection depends on coordinated action across healthcare systems, educational institutions, and community organizations. A central component is the design of interventions that account for health disparities and incorporate cultural responsiveness.

This review adopts a narrative design to integrate evidence across disciplines that often operate separately—neurosurgery, public health, and early childhood education. A narrative approach was chosen because the goal was conceptual synthesis rather than exhaustive enumeration of studies or effect-size estimation. This method allows for broader interpretation of diverse evidence sources, including clinical studies, educational interventions, and implementation analyses, which may not conform to the strict inclusion or outcome criteria required in systematic or scoping reviews.

## 2. Materials and Methods

### 2.1. Literature Search Strategy

A narrative review examined the relationship between early childhood health education and the prevention of neurosurgical conditions. The review covered publications from January 2000 through August 2025. Five major electronic databases were searched: ERIC, PsycINFO, MEDLINE, CINAHL, and Education Source—search terms combined descriptors for populations, educational interventions, neurosurgical conditions, and outcome measures. To ensure coverage of specialty literature, targeted searches were also conducted in neurosurgical journals and databases, including Neurosurgical Focus, Journal of Neurosurgery: Pediatrics, and The Lancet Global Health.

Search terms were combined using Boolean operators (AND, OR) to capture the intersection of early childhood education, preventive health interventions, and neurosurgical outcomes. The search syntax was adapted for each database to account for variations in indexing and subject headings, ensuring comprehensive coverage across both medical and educational sources.

Search terms were organized around three groups of preventable conditions. For TBI, the terms used were “safety education”, “helmet use”, “fall prevention”, and “playground safety”. For hydrocephalus, the terms were “infection prevention”, “early warning signs”, and “post-infectious complications”. For NTDs, the terms were “folate education”, “maternal nutrition”, and “prenatal counseling”. Reference lists of all included articles were also reviewed manually to identify additional studies.

Although this review used a systematic literature search to ensure comprehensive coverage across multiple databases, the synthesis itself followed a narrative approach. The systematic element refers to the transparent and replicable search strategy used to identify studies across ERIC, PsycINFO, MEDLINE, CINAHL, Education Source, and neurosurgical databases. After removing duplicates, titles and abstracts were screened for relevance to early childhood education, neurological health, and prevention of neurosurgical conditions. Full texts meeting inclusion criteria—studies involving children aged 2–8 years, preventive education, or neurological outcomes—were retained for qualitative analysis. The narrative synthesis was then structured using implementation science and ecological systems theory frameworks to group studies by condition, intervention type, and implementation strategy, and to identify cross-cutting preventive themes. This approach allowed for an integrative, conceptually driven understanding of how educational and clinical evidence intersect in preventing pediatric neurosurgical conditions.

### 2.2. Study Selection and Inclusion Criteria

We selected studies focusing on children aged 2–8 years in formal early childhood education settings that examined health education interventions related to neurological health, injury prevention, and early identification of neurological conditions. Titles and abstracts were first screened for relevance, followed by a full-text review of eligible articles. Reviewers independently evaluated study relevance based on predefined inclusion criteria, and disagreements were resolved through discussion until consensus was reached. This process ensured consistency and minimized selection bias during study identification. The review considered both empirical studies and conceptual papers to reflect the range of perspectives connecting educational practice with neurosurgical outcomes. No restrictions were placed on study design. Clinical studies, educational interventions, epidemiological analyses, qualitative research, and implementation studies were incorporated without imposing rigid criteria for methodological quality. This approach was chosen to allow the integration of innovative work that, while not always conforming to strict methodological standards in the medical or educational sciences, provided valuable insights into preventive and educational practices. In addition, policy documents, evaluations of educational curricula, and public health reports relevant to integrating neurological health content into early childhood education were also included. To ensure transparency in study selection, relevance was assessed based on clear alignment with the review objectives and the presence of measurable outcomes related to neurological health or prevention. Each study was reviewed for methodological clarity, sample description, and intervention documentation to gauge reliability. Although no formal quality scoring system was applied, studies lacking sufficient methodological detail or outcome data were excluded from synthesis. This approach balanced inclusivity across disciplines with an emphasis on empirical rigor and practical applicability to early childhood and neurosurgical prevention contexts.

### 2.3. Analytical Framework and Synthesis Approach

The analysis used a narrative synthesis informed by concepts from implementation science and ecological systems theory. Evidence was organized with matrices that linked educational interventions to neurosurgical outcomes, classified by condition, intervention type, and implementation strategy. The synthesis was carried out in three steps: (1) grouping studies by condition and educational focus, (2) identifying patterns and themes within and across groups, and (3) constructing frameworks that outlined possible prevention pathways. Bronfenbrenner’s Ecological Systems Theory and public health prevention models were applied to interpret how interventions function at the individual, family, organizational, and policy levels. Implementation science frameworks were also used to examine barriers, facilitators, and strategies for moving research into practice, focusing on cultural relevance and equity in intervention design.

The narrative synthesis followed a structured, multi-step process. Data from eligible studies were extracted into summary matrices that captured study objectives, population characteristics, intervention type, and reported outcomes. Themes were identified through repeated review of these matrices, focusing on recurring patterns in intervention design, implementation barriers, and measured effects. To enhance transparency, framework development drew on consensus between reviewers to ensure that identified categories reflected consistent patterns across studies rather than single observations. This process allowed for a reproducible organization of findings while maintaining the interpretive depth typical of narrative reviews.

## 3. Results

### 3.1. TBI Prevention Through Developmentally Sequenced Education

Studies show that prevention of TBI in children is most effective when teaching methods match the developmental stage rather than using the same approach for all ages. In preschool-aged children (3–5 years), play-based safety programs have been linked to lower injury rates. These programs work best when built into everyday curricula and delivered through activities such as guided play, safety drills, and practice with real-life scenarios [11,12]. These interventions demonstrate sustained improvements in teacher practices and child safety behaviors at 1-year follow-up, with programs initiated in early childhood showing higher knowledge retention compared to those initiated in elementary school [2,13].

A critical advancement in the field involves peer-led behavioral skills training models for early elementary students (*6–8* years). Participants in the Safety Ambassadors Program, which uses high school students as aspirational role models to teach younger kids about injury prevention, showed notable gains in their safety knowledge and behavior modification [14]. With older students acting as believable, captivating messengers, this method makes safety messages more salient than traditional adult-delivered instruction by utilizing children’s developing social learning and abstract thinking skills. The success of this model highlights how important it is to align educational delivery methods with developmental stages, adopting considerably different pedagogical methods in addition to content adaptation [15].

The devastating impact of TBI on educational outcomes provides compelling justification for prevention investment. All four areas of school readiness—early learning skills, self-regulation, social-emotional development, and physical health—show significant deficiencies in children with a history of TBI [3]. Because of this evidence, the discussion shifts from considering TBI prevention primarily as a medical intervention to acknowledging it as essential to educational parity. The failure to implement evidence-based prevention programs directly perpetuates cycles of educational disadvantage. It contributes to long-term health and economic disparities, even though preventable brain injuries effectively halve a child’s probability of kindergarten readiness (Table 1). These findings draw from studies that measured changes in safety behavior, cognitive readiness, and early learning outcomes following structured early-childhood safety education. The evidence consistently shows that when injury-prevention programs are matched to a child’s developmental stage, the benefits extend beyond immediate risk reduction to improved school readiness and behavioral regulation. The studies informing this synthesis were reviewed for the presence of measurable outcomes and follow-up data to ensure reliability. Together, this body of work demonstrates that developmentally sequenced education not only prevents injuries but also supports the broader developmental and educational goals of early childhood programs.

### 3.2. Post-Infectious Hydrocephalus Prevention Through Systematic Infection Control

Post-infectious hydrocephalus is the most preventable type of pediatric hydrocephalus. Prevention efforts focus on educating families to recognize neonatal illness early and strengthening infection control within healthcare facilities. Reviews of 156 implementation studies suggest adequate infection control can prevent up to 70% of healthcare-associated infections. Research in neonatal units has shown that barriers and facilitators to implementation are similar across different resource settings [16].

The most important obstacles, according to implementation science research, are structural (poor physical layouts, staff shortages, and unclear responsibilities), knowledge-related (unreliable access to supplies like gloves and alcohol rub), and resource-related (training gaps for clinical staff and families). On the other hand, relational ties (strong teamwork and peer support), a shared sense of urgency regarding infection prevention, and an innate desire to protect vulnerable newborns are the most effective facilitators. The two most commonly used and successful implementation strategies are evaluative (practice audits with feedback to clinical teams) and educational (training meetings for staff and families) [16].

In high-burden areas with inadequate healthcare infrastructure, community-based prevention models exhibit promise. Educational programs that promote hygiene and teach mothers to recognize neonatal illness have been shown to lower rates of post-infectious hydrocephalus. The most effective efforts have involved cooperation between early childhood centers and community health workers [5,6]. These strategies acknowledge that the broader care ecosystem, including primary healthcare providers, community health workers, and mothers as important protective agents, is part of effective prevention beyond hospital walls (Table 2) [17]. The conclusions in this section are based on a synthesis of multiple implementation studies that examined infection control in neonatal and pediatric settings. Data were reviewed across both high- and low-resource contexts to identify common barriers, facilitators, and outcome patterns. Although effectiveness varied by setting, structured infection-control programs consistently showed large reductions in preventable infections when training, auditing, and family engagement components were present. The synthesis emphasized context-specific adaptation rather than uniform intervention design, highlighting that local capacity, staffing, and resource availability shape implementation success. Together, these insights clarify that the potential for up to 70 percent prevention reflects aggregated outcomes across diverse health systems, with results strongest where systematic practices and community participation were sustained.

### 3.3. Neural Tube Defect Prevention Through Policy Integration and Cultural Responsiveness

According to the evidence, mandatory food fortification is the most equitable and effective way to prevent NTDs. However, there are still significant gaps in policy implementation around the world. Research on comparative effectiveness shows that for supplementation programs to provide preventive benefits comparable to fortification programs, adherence rates must be higher than 90%. This threshold is nearly impossible in most public health settings [7]. In the United States, once fortification of enriched cereal grain products was made mandatory, NTD rates dropped quickly and have remained low. The change prevents about 1300 cases yearly and saves more than $600 million in direct medical costs [8].

Persistent differences in NTD rates among particular populations demonstrate the importance of cultural responsiveness, even in nations with fortification programs. Because of structural flaws in the original 1998 fortification mandate that excluded corn masa flour, a staple food in many Hispanic cultures, Hispanic women in the United States continue to have lower median serum folate levels and a higher prevalence of pregnancies affected by NTDs than other groups [18]. Since most manufacturers have not embraced the practice, subsequent 2016 regulations permitting voluntary fortification of corn masa flour have largely failed to protect large segments of vulnerable populations [18].

Qualitative research indicates that effective cultural adaptation necessitates addressing structural and educational components. Hispanic women report wanting health information from providers in person—pamphlets and written handouts are less preferred. Some hold fatalistic beliefs about birth outcomes, which affect how prevention messages are received. Programs that speak to these views directly and respectfully result in more successful outcomes [18]. These findings demonstrate that successful NTD prevention requires coordinated structural interventions (mandatory fortification of culturally relevant foods) and culturally responsive education delivered through trusted community messengers (Table 1).

### 3.4. Multi-Sectoral Partnership Models and Implementation Strategies

Studies have found that when community groups, pediatric clinics, and early childhood programs work together, the results are stronger than when each sector acts alone. Pediatric primary care is critical because regular well-child visits give providers a direct way to reach families during key developmental periods [19]. Reach Out and Read, an evidence-based program that serves almost 5 million children in the United States each year, shows that it is possible to incorporate health promotion directly into healthcare settings by using strengths-based coaching techniques, anticipatory guidance, and the delivery of educational materials [19].

Evaluations of the Video Interaction Project show lasting effects on parenting practices and children’s social and emotional development, both of which are tied to school readiness. The program is built into pediatric visits, where providers coach parents on everyday interactions with their children [19]. These methods produce “braided” prevention strategies, significantly increasing the intervention dose and likely impact by reinforcing consistent messaging from multiple reliable sources through educational efforts across settings.

Implementation studies highlight several structural elements that contribute to the longevity of collaborations. These include regular communication, joint training, and systems for shared decision-making [20]. Some prevention work is done through networks that go beyond the family. In such cases, early childhood centers serve as community health hubs. The models that show the best results focus on building parental capacity for making informed choices and speaking up for their children’s neurological health, rather than only handing out information (Table 3) [20].

### 3.5. Quality Rating and Improvement Systems as Policy Levers

Practical tools for implementing evidence-based neuroprotection practices throughout early childhood education systems are Quality Rating and Improvement Systems (QRISs). According to an analysis of QRIS indicators in 41 states in the United States, 95% currently have at least one indicator related to fostering mental health and child development, with common standards encompassing staff training, parental involvement, and developmental monitoring [23]. Although only 24% of state QRISs currently require these, the review finds considerable improvement opportunities, such as more precise requirements for validated screening tools, active parent participation in screening procedures, and collaborations with child care health consultants [23].

A strategic policy approach to making prevention a non-negotiable part of high-quality early childhood education involves incorporating specific neuroprotection indicators into QRIS frameworks. Examples include the need for yearly professional development on identifying neurological warning signs, evidence-based TBI prevention curricula, and culturally relevant educational resources on periconceptional health. QRISs can act as potent policy levers promoting the broad adoption and long-term viability of neuroprotective practices by incorporating these standards into the fundamental definition of quality (Table 3) [23].

## 4. Discussion

This review demonstrates that focusing only on individual behavior is not enough.

Early education and community-based interventions serve as critical upstream mechanisms for preventing pediatric neurosurgical conditions by addressing risk factors long before clinical treatment is required. For example, structured play-based safety programs in preschool settings reduce TBI by teaching children practical safety behaviors such as fall prevention, safe play practices, and helmet use. Similarly, family-centered hygiene and neonatal infection–recognition programs in low-resource communities have been shown to reduce rates of post-infectious hydrocephalus through earlier care-seeking and improved infection control. In the case of NTDs, culturally responsive nutrition education and awareness campaigns promoting folic acid intake—particularly among populations whose staple diets lack fortified grains—have led to measurable reductions in affected pregnancies. Together, these approaches demonstrate how early educational and community engagement efforts can modify behavioral, environmental, and nutritional determinants that directly influence the burden of neurosurgical pathologies in children.

Effective prevention in pediatric neurosurgery through early childhood education needs approaches that work on multiple levels. These approaches should address immediate risks and the broader social conditions influencing health. Our analysis reveals that effective prevention strategies must operate simultaneously at the organizational, individual, and policy levels, with early childhood education serving as a platform for strategic integration rather than merely a venue for curriculum delivery. The evidence indicates three critical insights that improve theory and practice in this area.

The pedagogical approach to safety education is just as crucial as the content itself, and its efficacy depends on delivery strategies that are developmentally appropriate and evolve as children’s social and cognitive skills develop. The shift from play-based experiential learning in preschool settings to peer-led behavioral skills training in the early elementary years highlights the need for intervention design to align with developmental neuroscience principles, particularly TBI prevention. However, critical gaps remain between TBI education and stage-specific intervention approaches [24,25]. The detrimental effects of early TBI on school readiness—injury history lowers the likelihood of kindergarten readiness from 42.4% to 22.4%—turn this from a medical condition into a severe crisis of educational equity that necessitates immediate investment in prevention [3]. This finding gives policymakers economic and equity-based reasons to support the prevention of TBI in early childhood. Framing prevention as cost-effective also links it to efforts to reduce long-term disparities. While research acknowledges the critical nature of the developmental stages for TBI, more collaborative and interdisciplinary research is needed to develop effective interventions [3].

For post-infectious hydrocephalus, the global burden concentrated in LMICs demands an upstream focus on preventing neonatal sepsis rather than expanding downstream surgical capacity. Interventions that work best focus on structural barriers, such as building layout and staffing, as well as resources like steady access to materials and training that involves staff and families. Efforts limited to changing individual behavior have been less effective. Implementation science research shows that effective infection prevention requires systematic attention to organizational factors [16]. Early childhood education has been included in strengthening the health system in some contexts. Programs often connect prenatal services with the work of community health workers. Neurological health is better protected in low-resource areas when the entire care system is reinforced, not just single services. PIF must be recognized as a condition of poverty with prevention efforts requiring the identification of high-prevalence regions, discovering responsible pathogens that are region specific, and the implementation of targeted public health interventions [26]. In Uganda, a unique strain of *paenibacillus thiaminolyticus* has been identified as a major post-infectious hydrocephalus contributor [27], and multimodal intervention suggestions include the reduction of healthcare-associated infections, chlorhexidine cord cleansing, and community-based antibiotic delivery [28]. More research is still required to identify definitive, scalable prevention models.

One example of how structural policy interventions have a bigger population impact than individual education is the prevention of NTDs. Supplementation approaches require unrealistic adherence rates to achieve comparable benefits, but the evidence clearly shows that mandatory food fortification is the most equitable and effective preventive strategy [7,8]. In fact, multiple studies demonstrate mandatory food fortification as more effective than voluntary programs. Two systematic reviews showed that mandatory folate fortification was more effective in NTD reduction with decreased hospitalization rates, increased infant survival rates [29], and greater cost-effectiveness [30]. Another systematic review of folic acid fortification in 193 counties showed 32% of the total population (69 countries) having mandatory fortification, 53% of the total population (47 countries) having voluntary fortification, and 15% of the total population (77 countries) having no fortification. Average plasma folate levels were higher in countries with mandatory fortification (36 nmol/L) compared to countries with voluntary (21 nmol/L) or no fortification (17 nmol/L), and NTD prevalence per 10,000 population was lower in countries with mandatory fortification (4.19) compared to voluntary fortification (7.61) and no fortification (9.66) [31]. Another study compared NTD rates within Hispanic and non-Hispanic populations and the implementation of a voluntary corn masa folate fortification program, but there were no significant NTD reductions in the Hispanic population, suggesting mandatory fortification as a more optimal preventive approach [32].

Structural interventions that overlook cultural dietary practices often fail to be effective. In the United States, disparities in NTD rates among Hispanic populations continue because corn masa flour was left out of the fortification policy [18]. This case suggests that reducing health disparities requires two key elements: education that reflects the cultural context and policies that address structural factors. Education must consider the different factors that shape health outcomes, and policies must address the broader context in which families live.

Studies have found that when early childhood programs, pediatric clinics, and community groups collaborate, the results are stronger than when each sector works independently. QRIS offers one way to incorporate prevention standards into early childhood programs. The Video Interaction Project demonstrates how pediatric visits can be utilized to coach parents in everyday interactions, thereby integrating educational strategies into healthcare settings [19,23]. These collaborations produce “braided” prevention strategies that address social determinants that individual programs cannot address independently. They also increase intervention dose and sustainability through consistent messaging from several reliable sources.

Most health promotion programs in early childhood settings have not clearly documented or evaluated how they were implemented. Implementation science frameworks can be utilized to address this gap and support the application of research in practice [21]. This gap makes it harder to repeat interventions and to learn why some fail. Hybrid effectiveness–implementation designs can mitigate this problem by outlining the implementation strategy upfront, assessing fidelity, and examining the context influencing results. Cultural responsiveness is part of what makes an intervention valid and should not be treated as optional. Cultural responsiveness should be treated as essential to the validity of an intervention, not as an optional adjustment. Research shows that when applied to majority populations, systematic cultural adaptation using linguistic, sociocultural, and constituent-involving strategies produces effect sizes comparable to those of the original interventions [22]. One particularly qualitative study of 26 Hispanic women highlights the importance of having targeted and culturally sensitive intervention approaches for neural protection. Considering programs beyond knowledge increase and fortification are warranted, given their specific cultural identities, beliefs, and norms [18].

### Implementation in Low-Resource Settings

In low-resource settings, implementing multilevel prevention strategies requires phased, context-specific integration rather than large-scale structural reform. At the individual level, programs can prioritize caregiver and teacher training using low-cost, play-based safety and hygiene modules adaptable to local languages and cultural norms. At the organizational level, early childhood centers and community health posts can function as shared platforms for delivering both educational and health messages, leveraging existing infrastructure without major financial investment. At the policy level, partnerships with non-governmental organizations, ministries of health, and local education authorities can facilitate incremental adoption of evidence-based practices by embedding them into ongoing maternal–child health and early learning initiatives. This stepwise, resource-sensitive approach allows multilevel prevention to be achieved through collaboration, capacity building, and sustainable integration into existing community systems.

While this review integrates evidence from multiple disciplines and regions, certain limitations should be acknowledged. The analysis may be influenced by potential selection bias because only studies published in English and indexed in major academic databases were included. Additionally, variation in methodological rigor and outcome measures across educational, clinical, and public health studies introduces heterogeneity that limits direct comparability. Evidence from low- and middle-income countries also remains sparse, particularly regarding scalable community-based neurosurgical prevention models. These constraints highlight the need for more standardized, globally representative research to guide the adaptation of early educational and community interventions in diverse cultural and healthcare contexts.

## 5. Conclusions

When interventions are created using implementation science principles, address social determinants of health, and use culturally sensitive methods, early childhood education offers a strategic and underutilized platform for preventing pediatric neurosurgical conditions. Instead of relying solely on single-setting interventions, the most effective prevention strategies emphasize upstream prevention through coordinated, multi-sectoral partnerships and combine individual education with structural policy changes. Future studies should focus on hybrid effectiveness–implementation designs with longitudinal outcome measurement to improve the evidence base for population-level prevention programs. By implementing these evidence-based strategies, early childhood education systems can promote health parity and considerably lessen the burden of avoidable neurosurgical conditions.

The significance of this review lies in its integration of evidence from neurosurgery, early childhood education, and public health to identify a unified preventive framework for pediatric neurological conditions. By linking developmental pedagogy with neurosurgical prevention, this study provides a novel interdisciplinary perspective that highlights education as a viable, underutilized pathway to improve neural outcomes in children. The synthesis demonstrates that effective early interventions—whether through safety education, infection prevention, or nutritional awareness—can substantially reduce the global burden of traumatic brain injury, post-infectious hydrocephalus, and neural tube defects. These findings underscore the need for sustained collaboration between educators, clinicians, and policymakers to embed neuroprotective strategies into early childhood systems worldwide, promoting lifelong neurological health and reducing preventable neurosurgical morbidity.

## Figures and Tables

**Table 1:** Prevalence and Preventability of Major Pediatric Neurosurgical Conditions.

Condition	Global Prevalence	Annual Impact in North America	Preventability	Key Prevention Strategies
**Traumatic Brain Injury**	2.2% of children in the United States annually [1]; higher rates than adults	69,000+ traumatic brain injury-related deaths in the United States in 2021; approximately 190 daily [1]	40–65% of pediatric cases are potentially preventable [1]	Developmentally sequenced safety education [11,12]; play-based learning (ages 3–5); peer-led behavioral skills training (ages 6–8) [14]; helmet use; playground safety standards [15]
**Post-Infectious Hydrocephalus**	1:1000 children worldwide [4]; 180,000 new cases annually, primarily in low- and middle-income countries [4]	An estimated 125,000 shunt procedures are performed annually [4]	Up to 70% of cases are preventable through infection control [16]	Systematic infection prevention and control in healthcare settings [16]; maternal education on neonatal illness recognition [5,6], community health worker training [17]; improved hygiene practices [5]
**Neural Tube Defects**	1–2:1000 live births globally [8]; 27.4:10,000 in regions without fortification [7]	Approximately 3000 affected pregnancies annually in the United States [8]	50–70% preventable with adequate folic acid intake [8,9]	Mandatory food fortification (most effective) [8,9]; culturally responsive periconceptional education [18]; supplementation programs [7]; preconception health promotion [8]

**Table 2:** Evidence-Based Educational Approaches by Developmental Stage and Setting.

Target Population	Intervention Approach	Key Components	Implementation Setting	Effectiveness Evidence
**Preschool Children (3–5 years)**	Play-based safety education [11,12]	Experiential learning; safety simulations; environmental modifications; curriculum integration [11,12]	Early childhood education centers	Sustained improvements in teacher practices and child safety behaviors at 1-year follow-up [2,13]
**Elementary Children (6–8 years)**	Peer-led behavioral skills training [14]	High school mentors as role models; interactive demonstrations; social learning emphasis [14,15]	Schools with cross-age programs [14]	Significant improvements in safety knowledge and behavior modification [14]
**Maternal/Infant Dyads**	Community-based infection prevention [5,6]	Hygiene education; neonatal illness recognition; treatment-seeking promotion [5,16]	Community health settings; early childhood centers [5,17]	Significant reductions in post-infectious hydrocephalus rates in intervention areas [5,6]
**Women of Reproductive Age**	Culturally responsive nutrition education [18]	Folic acid supplementation; food fortification awareness; preconception planning [8,18]	Healthcare settings, community centers, and early childhood education outreach [18,19]	Increased supplementation adherence; reduced neural tube defect rates in targeted populations [8,18]
**Healthcare Staff and Families**	Systematic infection control training [16]	Hand hygiene protocols; sterile procedures; equipment management; family involvement [16]	Neonatal intensive care units [16]	Up to 70% reduction in healthcare-associated infections [16]

**Table 3:** Implementation Framework for Sustainable Neurosurgical Prevention Programs.

Implementation Domain	Essential Components	Recommended Strategies	Evaluation Metrics	Policy Integration Opportunities
**Educator Preparation**	Neurological health knowledge; developmental pedagogy; prevention implementation skills [11,12,13]	Pre-service training modules; ongoing professional development with coaching; peer support networks [21,22]	Knowledge assessments; classroom observation; implementation fidelity measures [21]	Integration into early childhood education credentialing requirements [23]
**Family and Community Engagement**	Culturally responsive outreach; bi-directional communication; practical support provision [18,22]	Community-based participatory design; adapted materials; peer support programs; resource provision [20,22]	Participation rates across demographics, knowledge gains, and health-seeking behavior changes [22]	Inclusion in family engagement standards for licensed programs [23]
**Cross-Sector Partnerships**	Healthcare-education coordination; community resource integration; shared professional development [19,20]	Formal liaison models; integrated screening systems; joint training programs; referral networks [19,20]	Partnership sustainability measures; communication effectiveness; service coordination outcomes [19,20]	Requirements for health consultant partnerships in Quality Rating systems [23]
**Quality Assurance**	Evidence-based curriculum standards; continuous improvement processes; outcome monitoring [21,23]	Integration into Quality Rating and Improvement Systems; performance-based funding; technical assistance delivery [21,23]	Child knowledge and behavior assessments; injury incidence tracking; program quality indicators [21,23]	State-level policy requirements for prevention standards in early childhood programs [23]

## Data Availability

Data sharing is not applicable to this article as no new data were generated or analyzed in this study.
